# Transcriptional Regulation of Tetrapyrrole Biosynthesis in *Arabidopsis thaliana*

**DOI:** 10.3389/fpls.2016.01811

**Published:** 2016-12-01

**Authors:** Koichi Kobayashi, Tatsuru Masuda

**Affiliations:** Graduate School of Arts and Sciences, The University of TokyoTokyo, Japan

**Keywords:** *Arabidopsis thaliana*, chlorophyll, chloroplast, gene expression, heme, photosynthesis, porphyrin, tetrapyrrole

## Abstract

Biosynthesis of chlorophyll (Chl) involves many enzymatic reactions that share several first steps for biosynthesis of other tetrapyrroles such as heme, siroheme, and phycobilins. Chl allows photosynthetic organisms to capture light energy for photosynthesis but with simultaneous threat of photooxidative damage to cells. To prevent photodamage by Chl and its highly photoreactive intermediates, photosynthetic organisms have developed multiple levels of regulatory mechanisms to coordinate tetrapyrrole biosynthesis (TPB) with the formation of photosynthetic and photoprotective systems and to fine-tune the metabolic flow with the varying needs of Chl and other tetrapyrroles under various developmental and environmental conditions. Among a wide range of regulatory mechanisms of TPB, this review summarizes transcriptional regulation of TPB genes during plant development, with focusing on several transcription factors characterized in *Arabidopsis thaliana*. Key TPB genes are tightly coexpressed with other photosynthesis-associated nuclear genes and are induced by light, oscillate in a diurnal and circadian manner, are coordinated with developmental and nutritional status, and are strongly downregulated in response to arrested chloroplast biogenesis. LONG HYPOCOTYL 5 and PHYTOCHROME-INTERACTING FACTORs, which are positive and negative transcription factors with a wide range of light signaling, respectively, target many TPB genes for light and circadian regulation. GOLDEN2-LIKE transcription factors directly regulate key TPB genes to fine-tune the formation of the photosynthetic apparatus with chloroplast functionality. Some transcription factors such as FAR-RED ELONGATED HYPOCOTYL3, REVEILLE1, and scarecrow-like transcription factors may directly regulate some specific TPB genes, whereas other factors such as GATA transcription factors are likely to regulate TPB genes in an indirect manner. Comprehensive transcriptional analyses of TPB genes and detailed characterization of key transcriptional regulators help us obtain a whole picture of transcriptional control of TPB in response to environmental and endogenous cues.

## Introduction

**FIGURE 1:**
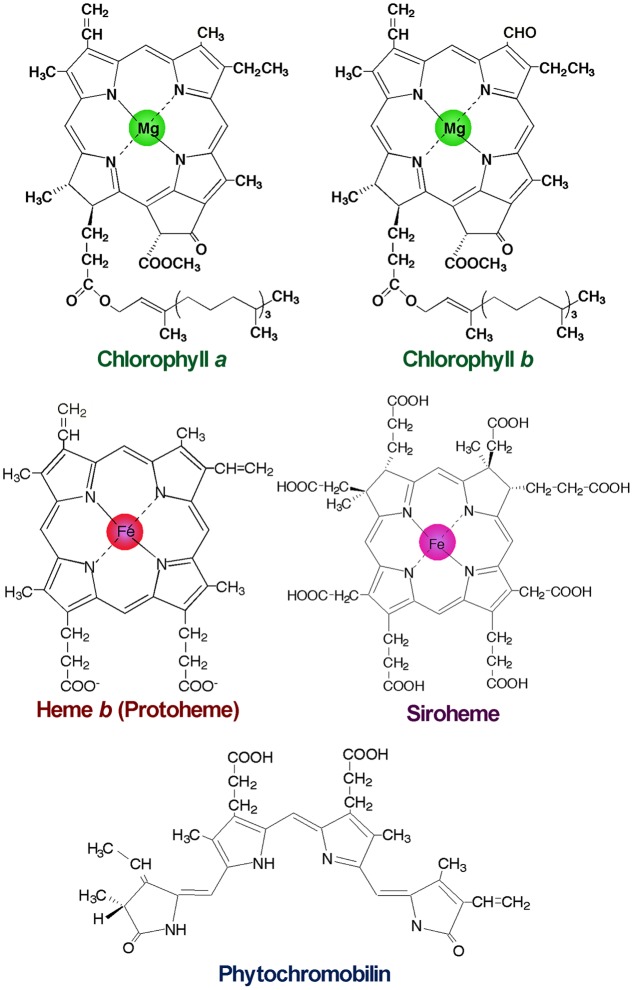
**Structures of major tetrapyrroles in plants.** Chlorophylls contain magnesium (Mg) for the central metal, whereas hemes and siroheme contain iron (Fe). Phytochromobilin is a linear tetrapyrrole functioning as a chromophore of phytochromes.

The development of photosynthetic machinery in chloroplasts is strictly regulated in response to various developmental and environmental cues to achieve efficient photosynthesis while avoiding photodamage (Jarvis and López-Juez, 2013). Particularly, the entire process of chlorophyll (Chl) biosynthesis should be strictly organized during chloroplast biogenesis because most Chl intermediates readily generate singlet oxygen and toxic radicals under light and consequently damage cells (Triantaphylidès and Havaux, 2009). In addition, Chl biosynthesis shares the common biosynthetic pathway with other tetrapyrroles such as heme, siroheme, and phycobilins (**Figure 1**), and metabolic flux to other tetrapyrroles affects Chl biosynthesis in plant cells. To produce Chl efficiently and safely on demand, plants utilize multiple levels of regulation including fine-tuning of enzymatic activities by cofactors, redox, and feedback systems, control of protein stability and suborganelle localization, and modulation of protein complex formation with regulatory proteins (Brzezowski et al., 2015; Kobayashi and Masuda, 2016). In addition, transcriptional regulation is a central system used to coordinate each step of tetrapyrrole biosynthesis (TPB) with the formation and maintenance of the photosynthetic machinery in response to developmental and environmental status. This review focuses on recent findings in the transcriptional regulation of TPB in *Arabidopsis thaliana*.

### Overview of Chl Biosynthesis in Plants

**FIGURE 2:**
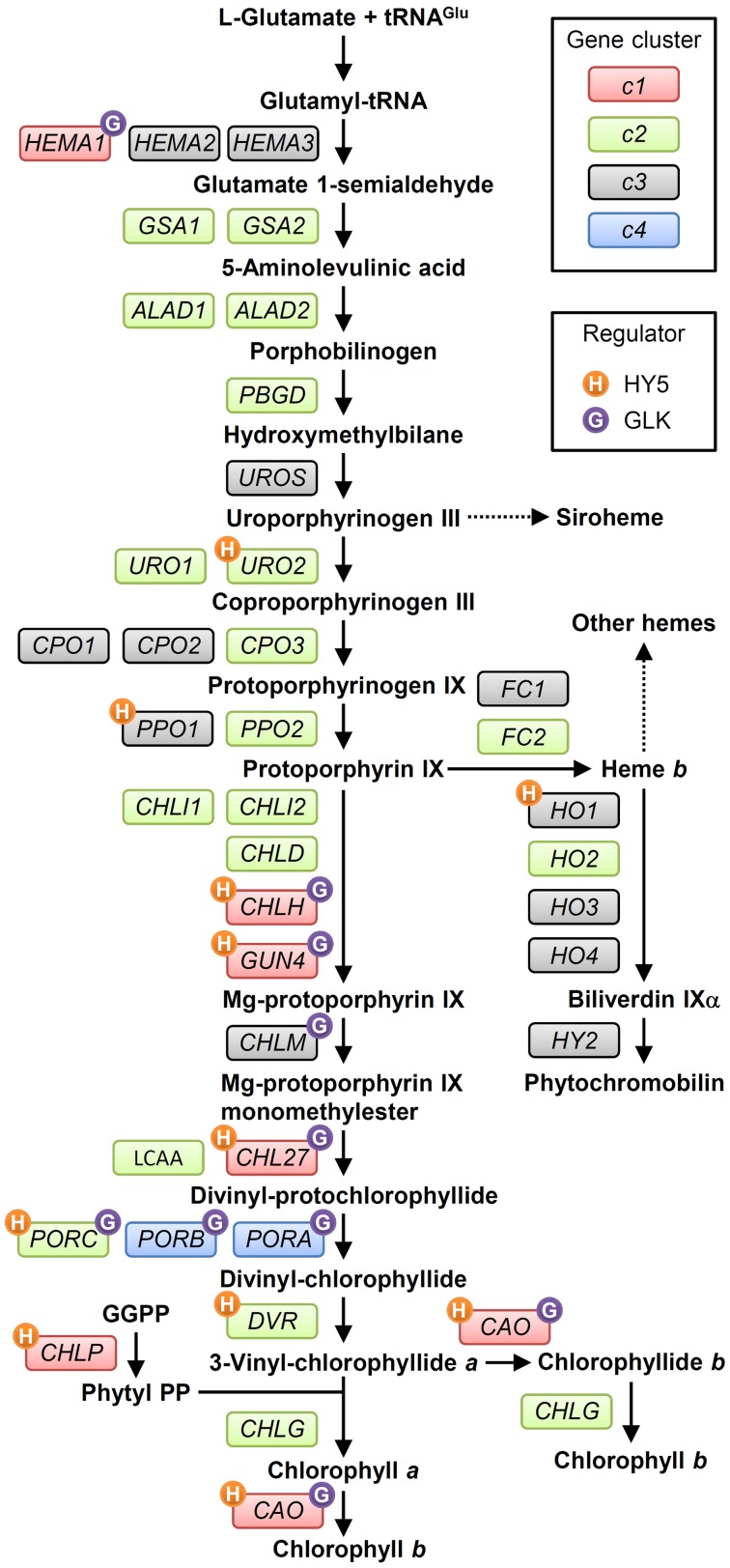
**The tetrapyrrole biosynthesis pathway (TPB) and TPB genes in *Arabidopsis*.** TPB genes are classified into four clusters (c1, c2, c3, and c4 in red, green, gray, and blue boxes, respectively) based on Matsumoto et al. (2004) and the ATTED-II coexpression database (Obayashi et al., 2007). Possible direct regulation of TPB genes by HY5 (Lee J. et al., 2007) and GLKs (Waters et al., 2009) is indicated by orange and purple circles, respectively, with initial letters of each regulator. Detail information for each gene is in Supplementary Table S1. GGPP, geranylgeranyl pyrophosphate; Phytyl PP, phytyl pyrophosphate.

In plants, all reactions of TPB take place in plastids. **Figure 2** shows the TPB pathway with the involved genes. For details, readers are referred to comprehensive reviews of TPB pathways (von Wettstein et al., 1997; Beale, 1999; Heinemann et al., 2008; Tanaka et al., 2011; Brzezowski et al., 2015; Kobayashi and Masuda, 2016). Chl shares the common biosynthetic pathway with other tetrapyrroles in the first multiple steps. Plants, algae and many bacteria synthesize 5-aminolevulinic acid (ALA), the universal precursor for all tetrapyrroles, from Glu via three enzymatic steps called the C_5_ pathway (Oborník and Green, 2005). Glu-tRNA^Glu^ formed from Glu and tRNA^Glu^ within plastids is reduced to Glu 1-semialdehyde (GSA) by Glu-tRNA reductase (GluTR). This reaction is the first committed and rate-limiting step of TPB (Papenbrock and Grimm, 2001). GSA is converted to ALA by GSA aminotransferase (GSAT), which catalyzes the intramolecular transfer of an amino group. Then two molecules of ALA are condensed asymmetrically to form the monopyrrole porphobilinogen (PBG), which is catalyzed by ALA dehydratase (ALAD; also known as PBG synthase). Four PBG molecules are then sequentially polymerized by PBG deaminase to result in the unstable linear tetrapyrrole hydroxymethylbilane (HMB). HMB is converted into the first macrocyclic tetrapyrrole, uroporphyrinogen (Urogen) III, by Urogen III synthase (UROS), then metabolized to coproporphyrinogen (Coprogen) III by Urogen III decarboxylase (UROD). A portion of Urogen III is also used for the synthesis of siroheme, the cofactor of nitrite and sulfite reductases involved in nitrogen and sulfur assimilation, respectively (Tanaka et al., 2011). Coprogen III is oxidized by Coprogen III oxidase (CPO) to form protoporphyrinogen (Protogen) IX, which is further oxidized by Protogen IX oxidase (PPO) to result in protoporphyrin (Proto) IX, the precursor for the synthesis of Chl and heme.

After the formation of Proto IX, the TPB pathway branches into two distinct pathways, namely the Mg- and Fe-branch for Chl and heme biosynthesis, respectively. In the Fe-branch, an Fe^2+^ ion is inserted into the Proto IX macrocycle by ferrochelatase to form protoheme (heme *b*), some of which is further converted into other hemes such as heme *a* and heme *c* or phytochromobilin, a linear tetrapyrrole functioning as a chromophore of phytochromes (**Figure 1**). In the Mg-branch, an Mg^2+^ ion is inserted into the Proto IX macrocycle in an ATP-dependent manner to form Mg-Proto IX. This reaction is catalyzed by Mg-chelatase consisting of the three subunits CHLD, CHIH, and CHLI in plants. GUN4, the regulatory protein of Mg-chelatase, assists this step by stabilizing the Mg-chelatase complex in membranes and mediating substrate and/or product channeling (Davison et al., 2005; Adhikari et al., 2011; Kopečná et al., 2015). Then a methyl group is added to Mg-Proto IX from S-adenosyl L-methionine by Mg-Proto IX methyltransferase, to result in Mg-Proto IX monomethyl ester (Mg-Proto ME). Mg-Proto ME is converted to 3,8-divinyl-protochlorophyllide (DV-Pchlide) by Mg-Proto ME cyclase, which forms a fifth isocyclic ring. Mg-Proto ME cyclase in plants is assumed to consist of three subunits including the membrane-bound subunits CHL27 (Rzeznicka et al., 2005) and LOW CHLOROPHYLL ACCUMULATION A (LCAA) (Albus et al., 2012; Hollingshead et al., 2012). After the reaction by Mg-Proto ME cyclase, the D ring of DV-Pchlide is reduced by Pchlide oxidoreductase (POR) to form 3,8-divinyl-chlorophyllide (DV-Chlide). Two different types of PORs, light-dependent LPOR and light-independent (dark-operative) DPOR, have been identified in photosynthetic organisms. Angiosperms possess only LPOR, which absolutely requires light for catalysis, whereas other plants, algae, and cyanobacteria possess both types of POR. Therefore, unlike other plants and cyanobacteria, angiosperms cannot synthesize Chl in darkness but instead accumulate Pchlide. DV-Chlide is converted to 3-vinyl Chlide *a* (MV-Chlide *a*) by DV-Chlide reductase (DVR). In addition, a geranylgeranyl chain is reduced by geranylgeranyl reductase encoded by *CHLP* to form a phytyl chain. Chl *a* is formed by esterification of MV-Chlide with geranylgeraniol or phytol, which is catalyzed by Chl synthase. Some of Chlide *a* or Chl *a* is converted into Chlide *b* or Chl *b* by Chlide *a* oxygenase (CAO) to diversify light-absorbing pigments in photosynthetic antenna complexes.

### Genes Involved in TPB and Their Expression Profiles in *Arabidopsis*

Characterization of the photosynthetic gene cluster in *Rhodobacter* species of purple bacteria brought the first detailed understanding of genes involved in TPB (TPB genes) (Young et al., 1989; Suzuki et al., 1997). In plants, pioneering biochemical and genetic works in barley and tobacco substantially contributed to reveal TPB pathways and the involved genes (von Wettstein et al., 1997; Beale, 1999). Subsequently, almost all TPB genes have been identified and extensively characterized in *Arabidopsis* (listed in **Figure 2**; Supplementary Table S1). In angiosperms, all TPB genes reside in the nucleus, whereas in other plant phyla, *chlL*, *chlN*, and *chlB* genes encoding DPOR subunits, which are missing in angiosperms, reside in the plastid genome (Fujita, 1996). In addition, tRNA^Glu^, the starting material for all TPB, is plastid-encoded. Most TPB enzymes or subunits in *Arabidopsis* form small families encoded by paralogous genes.

*Arabidopsis* has two paralogous genes for GluTR isoforms, namely *HEMA1* and *HEMA2*, and two genes for ferrochelatase isoforms, *FC1* and *FC2*. Another GluTR paralog, *HEMA3*, is probably a pseudogene (Matsumoto et al., 2004). *HEMA1* and *FC2* are actively transcribed in green tissues (Ilag et al., 1994; Chow et al., 1998) and play major roles in photosynthesis (Kumar and Söll, 2000; Scharfenberg et al., 2015; Woodson et al., 2015). *HEMA2* and *FC1* are expressed in non-photosynthetic tissues and induced under several stress conditions, which reflects their roles besides photosynthesis (Nagai et al., 2007). *Arabidopsis* also features a pair of genes for GSAT, ALAD, and UROD enzymes, but functional differences between each isoform remain elusive. For the conversion from Coprogen III to Proto IX via Protogen IX, three *CPO* and two *PPO* paralogs have been identified; mutant analysis showed that *CPO1* (Ishikawa et al., 2001) and *PPO1* (Molina et al., 1999) are presumably the main isoforms for each reaction. Phylogenetic analysis revealed that *PPO2* is unique to land plants and shares homology with *PPO* in green non-sulfur bacteria (Kobayashi et al., 2014b), which suggests a unique role of this gene in land plants. The CHLI subunit of Mg-chelatase is also encoded by two isoforms: *CHLI1* plays a major role in photosynthesis (Koncz et al., 1990; Rissler et al., 2002), whereas *CHLI2* has an auxiliary role in the assembly of the Mg-chelatase complex (Kobayashi et al., 2008). In addition, *Arabidopsis* LPOR isoforms are encoded by *PORA*, *PORB*, and *PORC*, whose expression is differentially regulated in response to light, described later. For the synthesis of a linear tetrapyrrole biliverdin IX from heme *b*, four heme oxygenase (HO) genes have been identified, with *HO1* playing a major role in the reaction (Davis et al., 1999; Muramoto et al., 1999).

**FIGURE 3:**
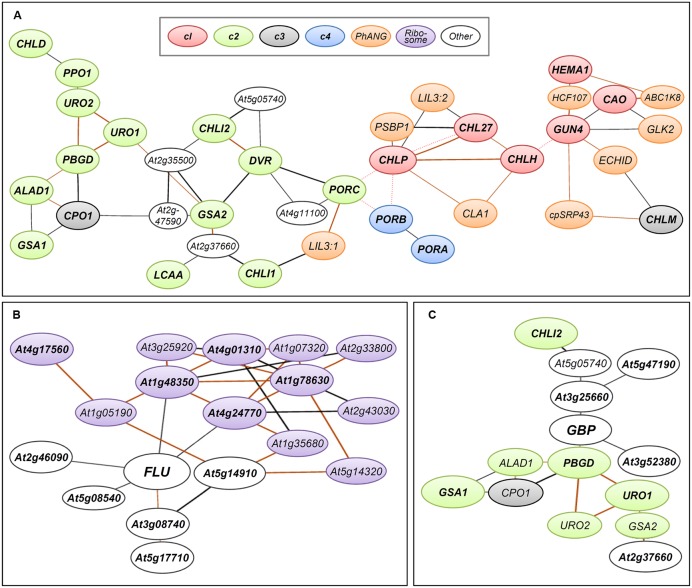
**Coexpression networks of *Arabidopsis* genes involved in TPB.** Coexpression networks of **(A)** TPB genes in cl (red), c2 (green), c3 (gray), and c4 (blue) clusters, **(B)** FLUORESCENT IN BLUE LIGHT (FLU, At3g14110), and **(C)** GluTR-binding protein (GBP, At3g21200) formed with photosynthesis-associated nuclear genes (PhANGs, orange), plastid ribosome-related genes (purple) and other nuclear genes (white). The coexpression networks were drawn by using the NetworkDrawer of the ATTED-II database v8.0 (Aoki et al., 2016; http://atted.jp/) with “add a few genes” and “Draw PPIs” options. Query genes are **(A)** all TPB genes listed in Supplementary Table S1, **(B)** *FLU* and its top 10 coexpressed genes (Supplementary Table S2) and **(C)** *GBP* and its top 10 coexpressed genes (Supplementary Table S3). Orange lines indicate highly reliable coexpression that is repeatedly detected in multiple coexpression data sets. Red dotted lines show links at protein–protein interaction levels. Query genes are in bold. Locus codes of PhANGs: *ABC1K8*, At5g64940; *CLA1*, At4g15560; *cpSRP43*, At2g47450; *ECHID*, At1g60550; *GLK2*, At5g44190; *HCF107*, At3g17040; *LIL3:1*, At4g17600; *LIL3:2*, At5g47110; *PSBP1*, At1g06680.

To monitor the expression of TPB genes during seedling development in *Arabidopsis*, Matsumoto et al. (2004) conducted a small-scale transcriptome analysis covering most TPB genes. The authors found that TPB genes can be classified into four clusters (c1–c4) based on their expression profiles in response to light and the circadian clock. The c1 cluster contains *HEMA1*, *CHLH*, *CHL27*, and *CAO*, whose expression is repressed in dark-grown seedlings but rapidly induced by light together with *LHCB6*, which encodes a subunit of light-harvesting complex II. During photoperiodic seedling growth, these genes show well-synchronized oscillation together with *LHCB6* under diurnal and circadian rhythms. Similarly, in tobacco, a rhythmic coexpression of *HEMA1* and *CHLH* with an *LHC* gene is linked with diurnal and circadian control of tetrapyrrole metabolism (Papenbrock et al., 1999). Genes of the cl cluster form a tight coexpression network with each other and additional TPB genes, *GUN4* and *CHLP* (**Figure 3A**) (Masuda and Fujita, 2008) in the ATTED-II coexpression database (Aoki et al., 2016). Thus, the c1 genes including *GUN4* and *CLHP* likely have a central role in regulating Chl biosynthesis as key TPB genes. Moreover, the key TPB genes are involved in a large coexpression network consisting of photosynthesis-associated nuclear genes (PhANGs) (Kobayashi et al., 2012b). Specifically, LIGHT-HARVESTING-LIKE 3 proteins (LIL3:1 and LIL3:2), which anchor the geranylgeranyl reductase encoded by *CHLP* to the membrane via direct interaction and facilitate its enzymatic activity (Tanaka et al., 2010; Takahashi et al., 2014), are involved in the coexpression network of TPB genes (**Figure 3A**). Thus, these genes may share a global transcriptional regulatory system to orchestrate Chl biosynthesis with the synthesis of cognate proteins and assembly of the photosynthetic apparatus during chloroplast biogenesis and maintenance.

The c2 cluster is the largest cluster and widely includes genes for reactions from the earlier to later steps of TPB (**Figure 2**). The expression of c2 genes is induced by light and oscillated with diurnal rhythm but with smaller amplitude than that of c1 genes. Unlike c1 genes, c2 genes are not under circadian control, so light may be the primary determinant for the c2 gene cluster during a photoperiodic cycle (Matsumoto et al., 2004). The c2 genes form a coexpression network within the cluster, which connects in part with the network of the c1 cluster (**Figure 3A**) (Masuda and Fujita, 2008). Thus, although c2 genes may not be the main regulators of TPB, their expression is likely coordinated with the c1 genes to avoid excess accumulation of photoreactive tetrapyrrole intermediates. The *LCAA* gene in *Arabidopsis*, which encodes a membrane subunit of Mg-Proto ME cyclase, is involved in a coexpression network with c2 genes (**Figure 3A**). Thus, this gene may belong to the c2 cluster, whereas another membrane subunit of Mg-Proto ME cyclase, *CHL27*, belongs to the c1 cluster.

Meanwhile, genes in the c3 cluster, many involved in heme metabolism, are not responsive to light and circadian rhythms, which suggests a minor contribution of these genes to the transcriptional regulation of TPB in such conditions (Matsumoto et al., 2004). As an exception, *CHLM* in the c3 cluster connects with the c1 genes in the coexpression network (**Figure 3A**), so *CHLM* may be coregulated with cl genes under some conditions.

The c4 cluster comprises only two genes, *PORA* and *PORB*, whose transcripts accumulate in dark-grown seedlings and rapidly decrease with illumination. This profile is consistent with the substantial accumulation of the Pchlide–LPOR complex within etioplasts in dark-grown cotyledons and rapid breakdown of the complex after illumination (Fujita, 1996). This profile is unique to angiosperm LPOR genes. Other plants and algae essentially have DPOR genes in addition to LPOR and therefore they rarely accumulate the Pchlide–LPOR complex in the dark (Fujita, 1996). In fact, *LPOR*s in gymnosperms and mosses are upregulated by light with other photosynthesis-associated genes (Takio et al., 1998; Skinner and Timko, 1999). In *Arabidopsis*, both *PORA* and *PORB* show circadian rhythmic expression, with an oscillation peak slightly delayed as compared to c1 genes. The expression of *PORA* is much lower than that of *PORB* under the light–dark cycle (Matsumoto et al., 2004) and almost undetectable in light-adapted mature seedlings (Armstrong et al., 1995; Oosawa et al., 2000; Su et al., 2001). Meanwhile, another *LPOR* in *Arabidopsis*, *PORC*, whose expression profile greatly differs from that of *PORA* and *PORB* (Oosawa et al., 2000; Su et al., 2001), is classified in the c2 cluster with its light-inducible and diurnal cycle-dependent characteristics. Thus, circadian-regulated *PORB* and diurnal-regulated *PORC* may fine-tune Chl biosynthesis together with other TPB genes after greening, as suggested by the strong growth defects of *porB porC* double mutant during photoperiodic growth (Frick et al., 2003). Because *PORA* can complement the *porB porC* double mutant with its ectopic overexpression (Paddock et al., 2010), the different physiological roles among the POR isoforms are mainly due to their specific expression patterns. This notion is consistent with the fact that some angiosperms possess only a single *POR* gene in their genome (Spano et al., 1992; Fusada et al., 2000).

In addition to the enzymes involved in TPB, posttranslational regulatory proteins also strongly affect TPB pathways. FLUORESCENT IN BLUE LIGHT (FLU), which is a negative regulator of ALA synthesis, interacts with *HEMA1*-encoded GluTR1 to prevent excess accumulation of Chl precursors particularly Pchlide (Meskauskiene et al., 2001; Meskauskiene and Klaus, 2002; Goslings et al., 2004). The mRNA level of *FLU* is increased by light, whereas its protein level is constant during seedling growth in the dark and after transfer to light (Goslings et al., 2004; Apitz et al., 2014). Because it is a negative regulator of TPB, *FLU* is not transcriptionally linked with TPB genes in the coexpression network (**Figure 3B**; Supplementary Table S2). Considering the substantial increase in *HEMA1* expression in response to light (Matsumoto et al., 2004), the increased ratio of GluTR1 to FLU during a dark-to-light transition would contribute to an increased capacity of the TPB pathway. Of note, FLU is coexpressed with many plastid ribosome-related genes (**Figure 3B**; Supplementary Table S2), so FLU may be somehow associated with protein synthesis in plastids.

A membrane bound GluTR-binding protein (GBP) is also involved in posttranslational regulation of GluTR activity. GBP interacts with GluTRs and recruits them to the thylakoid membrane (Czarnecki et al., 2011). Binding of GBP to the N-terminal domain of GluTR1 inhibits degradation of GluTR1 by the caseinolytic protease and allows for ongoing ALA synthesis for continuous heme formation (Apitz et al., 2016). Unlike *HEMA1* in the c1 cluster, *GBP* expression is almost unresponsive to light and the circadian clock and is similar to expression profiles of the c3 TPB genes (Czarnecki et al., 2011). Meanwhile, the expression profile of *GBP* in different tissues and developmental stages is similar to that of *HEMA1*. In the ATTED-II database (Aoki et al., 2016), *GBP* forms a coexpression network with many c2 genes involved in the common TPB pathway (**Figure 3C**; Supplementary Table S3). Because GBP assists heme formation by maintaining GluTR activity in the membrane, the coexpression of *GBP* with the c2 TPB genes may render the TPB pathway more efficient under varying developmental and environmental conditions.

### Transcriptional Factors Involved in TPB

#### LONG HYPOCOTYL 5 (HY5)

Because most TPB genes are light-inducible, the light signaling pathway plays a central role in the transcriptional regulation of Chl biosynthesis. HY5 is one of the pivotal transcription factors regulating many light-associated events including photomorphogenesis and chloroplast development downstream of photoreceptors (Bae and Choi, 2008). In the dark, the HY5 protein is ubiquitinated and destabilized through CONSTITUTIVE PHOTOMORPHOGENIC1 (COP1), a ubiquitin E3 ligase regulating the abundance of various light-signaling components (Lau and Deng, 2012). Upon light perception by photoreceptors, COP1 is inactivated; HY5 is accumulated in the nucleus and mediates multiple light responses.

A genome-wide chromatin immunoprecipitation-chip (ChIP-chip) analysis suggests that many TPB genes are direct targets of HY5 together with various PhANGs (**Figure 2**; Supplementary Table S1) (Lee J. et al., 2007). Only *HEMA1* in the cl cluster was not identified as the direct HY5 target in the ChIP-chip analysis. However, *HEMA1* expression in response to blue, red and far-red light was substantially decreased in a *hy5* mutant (McCormac and Terry, 2002), so *HEMA1* is also regulated by HY5. Loss of function of HY5 partially inhibits the *CHLH* expression and Chl accumulation in cotyledons during photomorphogenesis (Holm et al., 2002; Lee J. et al., 2007; Kobayashi et al., 2014a; Toledo-Ortiz et al., 2014); thus, this factor is important but not essential for Chl biosynthesis in leaf chloroplasts. Meanwhile, *hy5* roots exhibit an albino phenotype with very low expression of key TPB genes (Kobayashi et al., 2012a), which indicates that HY5 is particularly important for the expression of TPB genes in roots. In the *hy5* mutant, other factor(s) may compensate for the HY5 function in Chl biosynthesis in leaves but not roots.

HY5 specifically binds to the promoters of light-responsive genes through the G-box *cis*-element (CACGTG) (Chattopadhyay et al., 1998). ChIP-chip analysis showed significant enrichment of the G-box element in the promoter regions of HY5-targeted genes (Lee J. et al., 2007), which include key TPB genes and their coexpressed PhANGs (Kobayashi et al., 2012b). These data imply an importance of the G-box element for HY5-mediated transcriptional regulation of key TPB genes and associated genes. In fact, a deletion of the G-box sequence from the *CHLH* promoter region resulted in a decreased transcriptional activity particularly in the root (Kobayashi et al., 2012b). Moreover, Toledo-Ortiz et al. (2014) revealed that HY5 binds to G-box–containing promoter regions of *CHLH, PORC*, and other light-responsive genes. Thus, the interaction of HY5 with its target gene promoters on the G-box element would be associated with coordinated transcriptional regulation of key TPB genes with other PhANGs.

#### PHYTOCHROME-INTERACTING FACTORs (PIFs)

Most TPB genes are negatively regulated in the dark to avoid excess accumulation of Chl intermediates that cause photooxidation upon illumination. PIFs, the basic helix-loop-helix transcription factors involved in broad cellular processes as a signaling hub integrating light, hormone, and other multiple developmental signals (Leivar and Quail, 2011), play an essential role in repressing Chl biosynthesis in the dark. PIFs (PIF1, PIF3, PIF4, and PIF5) accumulate in the nucleus in the dark and repress photomorphogenic responses including Chl biosynthesis. Particularly, PIF1 acts as a cofactor of COP1 to synergistically degrade HY5 in the dark (Zhu et al., 2015). However, upon illumination, PIFs are phosphorylated and degraded via the ubiquitin–proteasome system in a phytochrome-dependent manner (Leivar and Quail, 2011).

Among six well-characterized PIF proteins, PIF1, PIF3, PIF4, and PIF5 mainly function redundantly to downregulate TPB genes during seedling growth in the dark. Loss of function of PIF1 and PIF3 caused increased expression of key TPB genes such as *HEMA1*, *CHLH*, and *GUN4* and excessive accumulation of Pchlide in the dark, which led to photobleaching of etiolated seedlings upon illumination (Huq et al., 2004; Stephenson et al., 2009). Etiolated seedlings of a *pif5* mutant also showed increased *CHLH* expression and photobleaching upon illumination (Shin et al., 2009). Furthermore, a quadruple mutant (*pifq*) lacking PIF1, PIF3, PIF4, and PIF5 showed global upregulation of TPB genes along with many PhANGs (Leivar et al., 2009; Shin et al., 2009). These data indicate that PIFs are required for the coordinated repression of TPB genes and other PhANGs in the dark.

PIF3 binds to G-box regions of *CHLH* and other PhANGs (Liu et al., 2013). The interaction of PIF1 with G-box regions of *CHLH*, *PORC* and *CAO* was also reported (Cheminant et al., 2011; Toledo-Ortiz et al., 2014). PIFs (PIF1 and PIF4) and HY5 antagonistically regulate these target genes presumably on the same G-box–containing region (Toledo-Ortiz et al., 2014). Liu et al. (2013) revealed that histone deacetylation of target gene promoters is involved in the regulation of PhANGs by PIF3. In the dark, HISTONE DEACETYLASE15 (HDA15) interacts with PIF3 and binds to the G-box regions of target gene promoters. HDA15 recruited to the target promoters decreases their transcriptional activities via histone deacetylation. However, in the light, PIF3 is degraded by phytochrome signaling and HDA15 is dissociated from its target promoters. At the same time, histone acetyltransferases involved in light-responsive gene expression such as TAF1/HAF2 and GCN5 may acetylate histones to upregulate PhANGs (Benhamed et al., 2006). HY5 is genetically associated with these histone acetyltransferases, so HY5 may be involved in histone acetylation of target genes by recruiting these acetyltransferases in response to light.

In addition, Feng et al. (2014) reported that PIFs are involved in light-dependent gene repositioning within the *Arabidopsis* nucleus. The authors found that the *CAB* locus consisting of *LHCB1.1*, *LHCB1.2*, and *LHCB1.3* located at the nuclear interior in the dark but translocated rapidly to the nuclear periphery upon illumination before full transcriptional activation. However, in the *pifq* mutant, the *CAB* locus was positioned at the nuclear periphery even in the dark. A similar phenomenon was observed in *cop1* and *de-etiolated 1* (*det1*) mutants. Thus, PIFs may play a role to retain the *CAB* locus in the nucleus interior in the dark with COP1 and DET1. In addition, other representative light-responsive gene loci including *CHLH* showed light-dependent reposition within the nucleus as observed in the *CAB* locus (Feng et al., 2014). Because these gene loci are separately distributed throughout the *Arabidopsis* genome, the light-dependent gene locus reposition is likely a common mechanism to induce PhANGs including key TPB genes in response to light.

### GOLDEN2-LIKE (GLK) Transcription Factors

The GLK gene family is involved in the transcriptional regulation of chloroplast biogenesis in diverse groups of land plants. Loss of function of GLKs perturbed chloroplast biogenesis in leaves (Hall et al., 1998; Fitter et al., 2002; Wang et al., 2013), whereas their gain of function in various plants enhances chloroplast development in non-photosynthetic organs such as roots and fruits in addition to leaves (Nakamura et al., 2009; Kobayashi et al., 2012a; Powell et al., 2012).

Many plant species have GLK genes in pairs (*GLK1* and *GLK2*) (Wang et al., 2013). In *Arabidopsis*, *GLK1* and *GLK2* are functionally equivalent, and only the double knockout mutant (*glk1 glk2*) showed perturbed chloroplast development with reduced expression of PhANGs in leaves (Fitter et al., 2002). Meanwhile, transient induction of exogenous *GLK*s in the *glk1 glk2* double mutant caused immediate upregulation of the PhANGs, especially those associated with Chl biosynthesis and light harvesting, which suggests that these primary inducible genes are direct targets of GLKs (Waters et al., 2009). Moreover, a ChIP analysis confirmed the direct binding of GLK1 to the promoter regions of its target genes including key TPB genes (Waters et al., 2009). With the exception of *PORA* and *PORB*, these TPB genes form a tight coexpression network with other GLK-targeted PhANGs (Masuda and Fujita, 2008; Kobayashi et al., 2012b), which implies an involvement of GLKs in the coexpression network of key TPB genes and PhANGs as central regulators. Of note, *GLK2* itself is also involved in the coexpression network with linking to the cl genes (**Figure 3**), which suggests a tight transcriptional coordination between *GLK2* and the target TPB genes.

Promoter analysis of GLK-targeted genes revealed a putative GLK-recognition *cis*-element (CCAATC) (Waters et al., 2009). The GLK-recognition element is significantly co-enriched with G-box in the promoter regions of key TPB genes and coexpressed PhANGs (Kobayashi et al., 2012b). *Arabidopsis* GLK1 and GLK2 can interact with G-box binding factors in yeast (Tamai et al., 2002). Moreover, enhanced Chl accumulation in roots by GLK overexpression required a G-box binding factor HY5 (Kobayashi et al., 2012a). Thus, GLKs may function in transcriptional regulation of Chl biosynthesis in collaboration with G-box binding factors such as HY5.

### Other Transcription Factors

GATA factors are transcriptional regulators that recognize a G-A-T-A core sequence of gene promoter regions. Of approximately 30 members of the GATA factor family, class B GATA factors (B-GATAs) are implicated in the regulation of Chl metabolism in addition to various developmental processes related to light and several hormonal responses (Behringer and Schwechheimer, 2015). Two B-GATAs, GNC (GATA, NITRATE-INDUCIBLE, CARBON METABOLISM INVOLVED) and GNL/CGA1 (GNC-LIKE/CYTOKININ-RESPONSIVE GATA TRANSCRIPTION FACTOR1), play a role in regulating chloroplast differentiation. Double-knockout mutations of GNC and GNL (*gnc gnl*) decreased Chl content in leaves, whereas overexpression of either gene enhanced ectopic Chl accumulation in leaf epidermis, roots and hypocotyls (Mara and Irish, 2008; Richter et al., 2010; Hudson et al., 2011, 2013; Chiang et al., 2012). The expression of *HEMA1*, *GUN4*, *PORB* and *PORC* was downregulated in the *gnc gnl* mutant but upregulated in the overexpressors of these factors (Hudson et al., 2011). However, in contrast to the direct regulation of TPB genes by HY5, PIFs, and GLKs, GNC and GNL appear to regulate these TPB genes in an indirect manner (Hudson et al., 2011). Four other B-GATAs (GATA15, GATA16, GATA17, and GATA17L) in *Arabidopsis* also function redundantly in the control of greening with GNC and GNL (Ranftl et al., 2016), and further studies are required for addressing the regulatory pathways of TPB gene expression by B-GATAs.

The transposase-derived transcription factors FAR-RED ELONGATED HYPOCOTYL3 (FHY3) and FAR-RED IMPAIREDRESPONSE1 (FAR1) are positive regulators of PHYA signaling and function in diverse developmental processes including Chl biosynthesis (Wang and Wang, 2015). Mutant analysis in *Arabidopsis* demonstrates that upregulation of *HEMA1* in response to red and far-red light stimuli requires FHY3 (McCormac and Terry, 2002). FHY3 is also important for the expression of *GUN4* and *CHLH* under far-red light (Stephenson and Terry, 2008). However, *HEMA1* but not *GUN4* and *CHLH* was identified as a putative direct target of FHY3 by ChIP-sequence analysis (Ouyang et al., 2011), which suggests that regulatory pathways by FHY3 are different between *HEMA1* and other genes (*GUN4* and *CHLH*). In addition, FHY3 and FAR1 induce the expression of *ALAD1/HEMB1* encoding an ALAD by binding to its promoter region through the FHY3/FAR1 binding site (CACGCGC) (Tang et al., 2012). The activity of FHY3 in the dark is partially repressed by PIF1 that physically interacts with the DNA binding domain of FHY3. FHY3 and FAR1 also physically interact with HY5 through their respective DNA binding domains (Li et al., 2010). The direct interaction between FHY3/FAR1 and HY5 affects PHYA signaling and the circadian clock (Wang and Wang, 2015), but its role in TPB gene regulation has not yet been elucidated.

The microRNAs miR171s (miR171a to c) and their targets scarecrow-like (SCL) transcription factors SCL6, SCL22 and SCL27 play an important role in transcriptional regulation of TPB genes, particularly those of the Chl pathway (Wang et al., 2010; Ma et al., 2014). Overexpression of miR171c enhanced degradation of the target *SCL* transcripts, which led to upregulation of *PORs* and *CAO* with increased Chl accumulation in leaves. A similar result was observed in the triple mutant of SCL6, SCL22, and SCL27. By contrast, overexpression of miR171-resistant SCL27 strongly decreased the expression of these TPB genes and Chl content in leaves. The data suggest that the balance between miR171s and their target SCLs strongly affect the expression of TPB genes involved in the Chl pathway in leaves. *In vivo* and *in vitro* analysis revealed that SCL27 can bind to the promoter of *PORC* through GT *cis*-element repeats and inhibit its expression (Ma et al., 2014). However, DELLA proteins, negative regulators of gibberellin signaling, physically interact with SCL27 and inhibit its DNA binding to the *PORC* promoter, which suggests an involvement of gibberellin signaling in TPB gene regulation via the DELLA–SCL27 interaction.

Xu et al. (2015) reported that a transcription factor REVEILLE1 (RVE1) binds to the promoter of *PORA* through a *cis*-element termed evening element (AAAATATCT) and upregulates *PORA* expression in the dark. Overexpression and loss of function of RVE1 increased and decreased the *PORA* expression in the dark, respectively. Moreover, RVE1 overexpression increased the greening rate of dark-grown seedlings with reduced reactive oxygen species (ROS) production and cell death, so RVE1 may be a crucial mediator of Chl biosynthesis and chloroplast development (Xu et al., 2015).

Key TPB genes of the c1 cluster are under transcriptional control by the circadian clock machinery (Matsumoto et al., 2004), in which TIMING OF CAB EXPRESSION1 (TOC1) plays a pivotal role as one of the core components. TOC1 functions as a transcriptional repressor by directly binding to the promoter of its target genes (Gendron et al., 2012), and *CHLH* is one of its regulatory targets. The transcript levels of *CHLH* increase in the day and decrease at night (Papenbrock et al., 1999; Matsumoto et al., 2004). However, silencing of TOC1 by RNAi induced *CHLH* expression in the night, and its overexpression suppressed *CHLH* expression in the day (Legnaioli et al., 2009). Moreover, TOC1 binding to the *CHLH* promoter was antiphasic to *CHLH* expression, which shows the direct suppression of *CHLH* expression by TOC1 to exert circadian oscillation.

Koussevitzky et al. (2007) reported that ABSCISIC ACID INSENSITIVE-4 (ABI4), an Apetala 2-type transcription factor, represses PhANG expression by binding to a specific *cis*-sequence (CCAC) in response to chloroplast dysfunction. Sun et al. (2011) proposed that the nuclear-localized N-terminal fragment of PTM protein, the chloroplast envelope-bound plant homeodomain transcription factor, is required for *ABI4* expression as a mediator of plastid-to-nucleus signaling. However, loss of ABI4 did not affect the *RBCS* promoter activity even when chloroplasts were disrupted by the photobleaching herbicide norflurazon (Acevedo-Hernandez et al., 2005). Moreover, the *abi4* mutant showed strong downregulation of *LHCB1.1* by norflurazon or by a plastidic translation inhibitor lincomycin as did the wild-type (Kerchev et al., 2011). Transcriptome analyses of the *abi4* mutant (Kerchev et al., 2011) and the ABI4 overexpression lines (Reeves et al., 2011) in *Arabidopsis* imply no direct involvement of ABI4 in TPB gene expression. Meanwhile, ABI4 is positively and negatively involved in the expression of a large number of genes associated with various cellular processes such as hormone and sugar signaling, redox homeostasis, and defense response, in addition to PhANGs (León et al., 2013). Specifically, ABI4 is required for downregulation of PhANGs under high glucose conditions (Arenas-Huertero et al., 2000; Acevedo-Hernandez et al., 2005). Moreover, ABI4 upregulates *PORA* presumably by inducing *COP1* expression in the dark (Xu et al., 2016). Therefore, multiple factors may work with ABI4 in a highly complex fashion under various conditions.

### Regulation of TPB Genes during Photomorphogenesis

**FIGURE 4:**
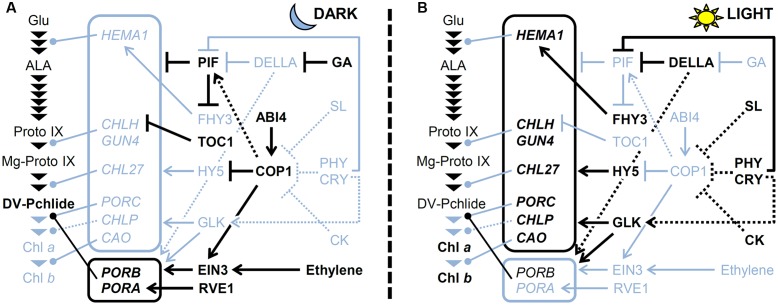
**A model for regulating TPB genes (A)** in the dark and **(B)** under light. The pathway for Chl biosynthesis is shown with important intermediates and enzymatic steps indicated by arrowheads. Key genes for the pathway shown in boxes are connected to each step that they involved. Arrows and bars represent positive and negative regulation, respectively. Dotted lines indicate indirect effects. Black and light blue colors represent activated and inactivated status, respectively. CK, cytokinin; CRY, cryptochrome; GA, gibberellic acid; PHY; phytochrome; SL, strigolactone.

Angiosperms germinated in darkness undergo a dark-adapted developmental program termed skotomorphogenesis, which is characterized by a prolonged hypocotyl and etiolated cotyledons containing dark-specific plastid etioplasts. In etioplasts, a substantial amount of LPOR is accumulated along with Pchlide *a* and NADPH to form prolamellar bodies (Sundqvist and Dahlin, 1997). In *Arabidopsis* seedlings, *PORA* and *PORB* are actively expressed during skotomorphogenesis (Armstrong et al., 1995; Matsumoto et al., 2004). Mutant analysis showed that the PORA and PROB proteins accumulated in etioplasts contribute to the formation of prolamellar bodies in an additive manner (Franck et al., 2000; Paddock et al., 2010). COP1 plays an essential role in this process (**Figure 4**), as suggested by a remarkable decrease in *PORA* and *PORB* transcripts with loss of prolamellar bodies in dark-grown *cop1* seedlings (Sperling et al., 1998). ABI4 is required for *PORA* expression in the dark, presumably with its activity to induce *COP1* expression (Xu et al., 2016). Moreover, the regulation of the *PORA* and *PORB* expression by COP1 involves ethylene signaling mediated by an ethylene-inducible transcription factor EIN3/EIL1. Ethylene and COP1 signaling stabilize the EIN3/EIL1 protein, which then upregulates *PORA* and *PORB* by binding to their promoter regions (Zhong et al., 2009). In addition, gibberellin signaling is involved in the regulation of *PORA* and *PORB* via DELLA proteins (Cheminant et al., 2011). DELLAs can upregulate *PORA* and *PORB* expression in the dark. Although DELLAs have a function to inhibit PIF activities by direct protein–protein interactions (de Lucas et al., 2008; Feng et al., 2008), they act on *PORA* and *PORB* expression independently of PIFs (Cheminant et al., 2011). In addition, RVE1 upregulates *PORA* but not *PORB* by directly binding to the *PORA* promoter (Xu et al., 2015). The relationship between RVE1 and other regulatory factors for *PORA* regulation in darkness remains unknown. Meanwhile, *PORC* expression is almost undetectable in etiolated seedlings (Oosawa et al., 2000; Su et al., 2001) and has no important role in the formation of prolamellar bodies (Frick et al., 2003; Masuda et al., 2003; Paddock et al., 2010).

With the exception of *POR*s actively transcribed in the dark, most TPB genes are repressed in dark-grown seedlings to prevent photodamage upon illumination (Matsumoto et al., 2004). As described earlier, PIFs play a central role in the negative regulation of TPB genes (**Figure 4**). Gibberellins accumulated in the dark contribute to the activation of PIFs by degrading DELLA proteins and releasing PIFs from inhibition by DELLAs (Cheminant et al., 2011). Meanwhile, major positive regulators involved in TPB gene expression are repressed in the dark. The HY5 protein is degraded in the dark via COP1/DET1-mediated ubiquitination (Osterlund et al., 2000). Moreover, the expression of *GLKs* and *B-GATAs* is low in the dark because they are light-inducible (Fitter et al., 2002; Manfield et al., 2007; Ranftl et al., 2016). Thus, activation of negative regulators and inactivation of positive regulators combined could keep the expression of key TPB genes low in the dark.

Upon illumination, Pchlide accumulated in the dark is immediately converted to Chlide by LPORs and then to Chl by Chl synthase. During this process, the expression of *PORA* and *PORB* is rapidly decreased in response to light, with possible involvement of degradation of EIN3 via inactivation of COP1 and ethylene signaling (Zhong et al., 2009). At the same time, *de novo* Chl biosynthesis is activated to develop functional chloroplasts in cotyledons. Both the phytochrome and cryptochrome photoreceptor families play a crucial role in upregulating key TPB genes such as *HEMA1*, *CHLH*, *GUN4*, and *CAO* (McCormac and Terry, 2002; Tepperman et al., 2006; Stephenson and Terry, 2008). Under light, phytochromes induce phosphorylation, ubiquitination, and subsequent degradation of PIFs (Leivar and Quail, 2011), which results in derepression of TPB genes. DELLAs also contribute to derepression of TPB genes by inhibiting PIF activities (Cheminant et al., 2011). Meanwhile, light mediates the accumulation of HY5 proteins by preventing COP1 activity and induces the expression of *GLKs* and *B-GATAs*, which together upregulate key TPB genes (**Figure 4**). In addition, cytokinin and strigolactone inhibit COP1 activity and increase HY5 protein level (Vandenbussche et al., 2007; Tsuchiya et al., 2010). In fact, upregulation of *HEMA1*, *CHLH* and *CHL27* in response to light was weakened in *Arabidopsis* cytokinin receptor mutants, whereas cytokinin treatment to dark-grown wild-type seedlings increased expression of these genes (Hedtke et al., 2012; Kobayashi et al., 2014a). Moreover, Cortleven et al. (2016) recently reported that type-B ARABIDOPSIS RESPONSE REGULATORs (ARRs) function in the greening process downstream of cytokinin signaling. In this process, ARR10 and ARR12 directly bind to the promoter regions of *HEMA1* in addition to *LHCB6* (Cortleven et al., 2016). These data suggest an importance of cytokinin signaling in upregulation of key TPB genes during photomorphogenesis. Melo et al. (2016) reported that in tomato, production of nitric oxide (NO) in response to light via phytochrome signaling promotes Chl accumulation presumably in part by downregulating negative regulators of photomorphogenesis such as *COP1* and *DET1* and upregulating *GLK1*. The authors proposed that negative and positive feedback loops between ethylene-NO and auxin-NO, respectively, are involved in regulating the greening of tomato seedlings (Melo et al., 2016).

### Circadian Regulation of TPB Genes

After establishing photoautotrophic growth with developed chloroplasts, plants maintain homeostatic Chl biosynthesis to support continued growth with high photosynthetic activity. Chl biosynthesis is coordinated with development of photosynthetic machinery by light and the endogenous clock, with concerted circadian regulation of key TPB genes with Chl-binding apoproteins during the day–night cycle playing a crucial role (Papenbrock et al., 1999; Matsumoto et al., 2004; Stephenson et al., 2009).

As described earlier, the core evening phase factor TOC1 decreases *CHLH* transcripts at night by directly binding to the *CHLH* promoter (Legnaioli et al., 2009). Abscisic acid treatment changes the circadian rhythm of *CHLH* transcripts by upregulating *TOC1* expression, although its relevance to regulation of Chl biosynthesis is unknown. A genome-wide ChIP-sequence analysis identified only *CHLH* as the TOC1 target among TPB genes (Gendron et al., 2012). Thus, regulatory pathways in response to the circadian clock differ between *CHLH* and other cl genes, although they all show very similar circadian oscillation (Matsumoto et al., 2004).

Together with TOC1, two morning-phased transcription factors, CIRCADIAN CLOCK-ASSOCIATED1 (CCA1) and LATE ELONGATED HYPOCOTYL (LHY), form a core transcriptional feedback loop to maintain circadian oscillation. CCA1 and LHY in *Arabidopsis* colocalize in the nucleus via physical interaction and function synergistically in regulating circadian oscillation (Lu et al., 2009). CCA1 and LHY bind to the same region (CCA1-binding site) of the promoter of *LHCB1.3* to activate it in response to light (Wang et al., 1997; Lu et al., 2009). The *LHCB1.3* promoter has a G-box element to bind HY5, in close proximity to the CCA1 binding site. CCA1 affects the binding of HY5 to *LHCB1.1* and *LHCB1.3* promoters via physical interaction. HY5 is required for regulating the circadian oscillation of the *LHCB1.1* transcripts (Andronis et al., 2008). Moreover, loss of HY5 decreased the peak expression of PhANGs including *CHLH* and *PORC* in daytime (Toledo-Ortiz et al., 2014). Thus, HY5 interacting with CCA1 plays an important role in the circadian regulation of at least some of the PhANGs including key TPB genes. Besides functioning in the output of light signaling, HY5 is involved in gating light signaling to the circadian machinery in collaboration with FHY3 and FAR1 (Li et al., 2011), which reflects a diverse function of HY5 in circadian regulation.

In contrast to HY5, PIFs function to downregulate key TPB genes during circadian oscillation. Stephenson et al. (2009) showed that the periodic expression of *HEMA1*, *CHLH* and *GUN4* observed during seedling growth in darkness was strongly disordered in *pif1* and *pif3* mutants with an increasing trend. The *pif1* and *pif3* mutations did not affect the circadian expression of *TOC1*, *CCA1* and *LHY* in dark-grown seedlings, so PIF1 and PIF3 function in the output from the circadian clock under such conditions (Stephenson et al., 2009). Moreover, the expression of *CHLH* and *PORC* was increased throughout circadian oscillation in the *pifq* mutant (Toledo-Ortiz et al., 2014). Thus, PIFs would function in circadian signaling to downregulate Chl biosynthesis with the development of photosynthetic machinery at night. TOC1 can interact with PIFs and represses their transcriptional activation activity (Soy et al., 2016). However, the effect of TOC1 on the function of PIFs as a transcriptional repressor of PhANGs needs to be addressed.

The expression of *GLK2* is regulated in a circadian-dependent manner, as is the expression of key TPB genes (Fitter et al., 2002). GLK2 is a strong inducer of key TPB genes, so oscillation of *GLK2* transcript levels could be associated with the circadian regulation of key TPB genes. *GNC* and *GNL* are also under circadian regulation with expression peak at pre-dawn (Manfield et al., 2007) and may affect the circadian oscillation of key TPB genes indirectly.

### Coordinated Regulation of TPB Genes with Chloroplast Functionality

**FIGURE 5:**
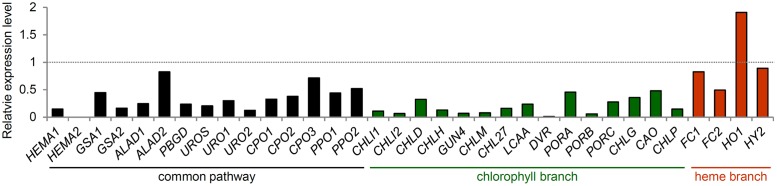
**Expression of TPB genes during leaf senescence.** Gene expression data in *Arabidopsis* were obtained from the AtGenExpress Visualization Tool (http://jsp.weigelworld.org/expviz/expviz.jsp) in the public transcriptome database. The expression of each gene in senesced leaves of 35-day-old plants was normalized to that in the sixth true leaves of 17-day-old seedlings (dotted line).

Plants need to coordinate Chl biosynthesis with the formation of the photosynthetic machinery to meet the variable demands for Chl during development and to prevent photooxidative damage by free Chl and its intermediates. Light induction of most TPB genes during chloroplast biogenesis is important to provide Chl for the newly synthesized photosynthetic apparatus. By contrast, the expression of most TPB genes is gradually decreased during plant maturation (Matsumoto et al., 2004) and strongly repressed during leaf senescence (**Figure 5**) (Lin and Wu, 2004). Such transcriptional regulation of Chl biosynthesis in coordination with chloroplast development is required to avoid photooxidative damage from free Chl and tetrapyrrole intermediates. In fact, deregulation of the TPB gene expression in *pif* mutants resulted in photobleaching of dark-grown seedlings with illumination (Huq et al., 2004; Leivar et al., 2009; Shin et al., 2009; Stephenson et al., 2009). Forced overexpression of *HEMA1* (Schmied et al., 2011) or *CHLH* (Shin et al., 2009) caused strong photooxidative damage to leaves in *Arabidopsis*. Moreover, a transient decrease in *CHL27* expression by dexamethasone-induced RNAi caused an imbalance in the metabolic flow of the Chl biosynthesis pathway and accumulation of Mg-Proto and Mg-Proto ME, which generates harmful ROS in the light (Schlicke et al., 2014). These data clearly show the importance of regulation of Chl biosynthesis at transcriptional levels.

The expression of key TPB genes is strongly associated with the biogenesis of the thylakoid membrane, where Chl forms complexes with photosynthetic proteins and other cofactors. The coordinated expression of TPB genes and other PhANGs with thylakoid membrane biogenesis was revealed in mutants defective in thylakoid lipid biosynthesis (Kobayashi, 2016). Impaired biosynthesis of galactolipids, the major lipid constituents of the thylakoid membrane, caused strong downregulation of TPB genes and other PhANGs concomitant with disrupted thylakoid lipid-bilayer formation (Kobayashi et al., 2013; Fujii et al., 2014). Similar results were observed in a mutant deficient in another major thylakoid lipid, phosphatidylglycerol (Kobayashi et al., 2015). However, in these lipid mutants, activation of thylakoid biogenesis via alternative lipid biosynthetic pathways by P starvation restored the decreased TPB gene expression (Kobayashi et al., 2013, 2015). By contrast, photosynthetic activity was still dysfunctional in these mutants, presumably because of lack of essential lipid molecules in photosynthetic complexes. These data suggest that TPB gene expression is coupled with the formation of the thylakoid lipid bilayer independent of photosynthetic activities. Moreover, the expression of TPB genes and galactolipid biosynthesis genes is coordinated with each other by light and cytokinin signaling (Kobayashi et al., 2014a), which may also contribute to the orchestration of Chl biosynthesis and thylakoid biogenesis.

Land plants are at constant risk of photooxidative damage in chloroplasts by fluctuating strong light and other biotic and abiotic stresses. Sudden exposure to high light and low temperature stresses, which cause over-reduction of the photosynthetic electron transport chain, quickly downregulates many TPB genes as well as PhANGs, presumably to minimize the potential of chloroplasts to generate ROS (Bode et al., 2016). Although the redox state of photosynthetic electron components has been implicated as a source of signals to regulate PhANGs (Oswald et al., 2001; Pfannschmidt, 2003), the mode of action remains unclear (Piippo et al., 2006; Bode et al., 2016). Although modification of Mg porphyrin levels by induced silencing of *CHLH*, *CHLM*, and *CHL27* did not immediately affect the expression of TPB genes and other PhANGs, the prolonged silencing of *CHL27* decreased the expression of some TPB genes along with PhANGs, presumably because of the generation of singlet oxygen (Schlicke et al., 2014). Moreover, a knockdown mutation of *Arabidopsis CHL27* by T-DNA insertion in its promoter region downregulated many TPB genes and PhANGs (Bang et al., 2008). These data suggest that modification of Mg porphyrin metabolism secondarily affects the transcriptional activity of TPB genes via ROS-mediated signaling.

When the chloroplast function is severely impaired, the expression of PhANGs, including most TPB genes, is shut off via the plastid-to-nucleus retrograde signaling. Several *Arabidopsis* mutants, with disrupted retrograde plastid signaling, have been identified and are named *genomes-uncoupled* (*gun*) mutants (Susek et al., 1993). In *gun* mutants, chloroplast dysfunction by herbicide or antibiotic treatments could not strongly decrease the expression of key TPB genes and other PhANGs (Strand et al., 2003; Moulin et al., 2008), which suggests that key TPB genes are under strict control of plastid signaling similar to other PhANGs. A plastidic pentatricopeptide repeat protein, GUN1, plays a central role in plastid signaling by integrating multiple plastid signals to shut off the expression of PhANGs in response to chloroplast dysfunction (Chi et al., 2013). Genetic evidence suggests that GUN1 signaling activates ABI4 to downregulate PhANGs in response to chloroplast dysfunction (Koussevitzky et al., 2007), although the significance of ABI4 in this process is controversial (Acevedo-Hernandez et al., 2005; Kakizaki et al., 2009; Kerchev et al., 2011). In addition, *GLK1* and *GLK2* are under transcriptional control by plastid signaling (Waters et al., 2009; Leister and Kleine, 2016). Furthermore, overexpressors of *GLK1* and *GLK2* showed a *gun* phenotype after norflurazon or lincomycin treatment (Leister and Kleine, 2016). GUN1 somehow downregulates *GLK1* expression in response to chloroplast dysfunction, which results in the downregulation of GLK targets including key TPB genes independent of ABI4 signaling (Kakizaki et al., 2009; Waters et al., 2009). Transcriptional regulation of GLKs in response to chloroplast functionality would help optimize Chl synthesis under varying environmental and developmental conditions.

Although HY5 is one of the central transcriptional activators of light-responsive genes, including many PhANGs, during photomorphogenesis as described earlier, Ruckle et al. (2007) showed that this factor represses its target genes via cryptochrome 1 (CRY1) when chloroplasts are dysfunctional. In the authors’ model, plastid signals convert HY5 from a positive to a negative regulator in a GUN1-independent manner and downregulate HY5-targeted genes. In fact, loss-of-function mutations in CRY1 or HY5 caused strong photodamage under high light conditions because of deregulation of PhANGs, and the additional *gun1* mutation to these mutants further enhanced the light-dependent growth defect (Ruckle et al., 2007). The data suggest that the dual transcriptional regulation of PhANGs by CRY1–HY5-mediated light signaling and GUN1 signaling is crucial for controlling the development of photosynthetic machinery according to light conditions and chloroplast functionality. We should note that *LHCB* genes are particularly subject to negative regulation by light signaling after lincomycin-induced plastid dysfunction and many other PhANGs including TPB genes showed only reduced light response with lincomycin treatment (Ruckle et al., 2012). Meanwhile, Kindgren et al. (2011) propose that HY5 is involved in the plastid signaling mediated by Mg-Proto IX. Feeding of Mg-Proto IX via roots strongly repressed *LHCB1.1* and *RBCS* expression in green tissues of wild-type *Arabidopsis* but not in the *hy5* mutant (Kindgren et al., 2011). Nonetheless, several biochemical and genetic studies have not supported the direct relationship between the expression of PhANGs and *in vivo* Mg-Proto IX levels (Mochizuki et al., 2008; Moulin et al., 2008; Terry and Smith, 2013; Schlicke et al., 2014), and the role of tetrapyrrole signaling in plastid signaling is still under debate.

### Regulation of TPB Genes in Response to Biotic and Abiotic Stresses

In addition to producing Chl, the TPB pathway is indispensable for providing heme as a protein cofactor or a regulatory molecule for various biological processes. *Arabidopsis HEMA1* and *FC2* play major roles in photosynthesis by being abundantly expressed in green tissues as described earlier, the expression of their counterparts *HEMA2* and *FC1* is low in these tissues but is strongly upregulated in response to ROS-generating treatments such as wounding, ozone, and herbicides (Nagai et al., 2007). Of note, the expression of *HEMA2* and *FC1* is increased in the *flu* mutant exposed to light after dark incubation (op den Camp et al., 2003). The *flu* mutant lacks the ability to regulate GluTR1 activity and therefore accumulates excess Pchlide in the dark (Meskauskiene et al., 2001). With light exposure, Pchlide accumulated in the mutant generates singlet oxygen, which subsequently induces a programmed cell death response mediated by plastid-localized EXECUTER proteins (Wagner et al., 2004; Lee K.P. et al., 2007; Zhang et al., 2014). These data suggest that *HEMA2* and *FC1* are also responsive to singlet oxygen signaling. Moreover, biotic stresses such as treatment with a bacterial elicitor flagellin 22 induce strong *FC1* expression and weak *HEMA2* expression (Scharfenberg et al., 2015; Espinas et al., 2016). Although oxylipin signaling mediated by jasmonic acid (JA) and 12-oxo phytodienoic acid (OPDA), produced via peroxidation reactions of membrane lipids, is deeply involved in wounding and biotic stress responses (Reinbothe et al., 2009), *FC1* and *HEMA2* are unresponsive to these oxylipins (Taki et al., 2005; Nagai et al., 2007), which is consistent with a report that JA and OPDA do not act as second messengers during singlet oxygen signaling (Przybyla et al., 2008). Because genes encoding the endoplasmic reticulum-localized cytochrome P450 family and cytosolic ascorbate peroxidase are co-upregulated with *HEMA2* and *FC1* in response to wounding and flagellin 22 treatment, the HEMA2-FC1 pathway may supply heme for extraplastidic factors associated with defensive functions (Nagai et al., 2007; Espinas et al., 2016). In fact, mutant analysis showed that FC1-producing heme is mainly allocated to extraplastidic organelles, which contrasts with the major role of FC2-producing heme in the chloroplast (Espinas et al., 2016). Differential regulation of paralogous genes in the same reaction step as observed between *HEMA1* and *HEMA2* as well as *FC2* and *FC1* would allow plants to coordinate the TPB pathway flexibly with various kinds of developmental conditions and biotic and abiotic stresses.

## Perspectives

Large-scale transcriptome analysis, targeted gene expression analysis and characterization of transcription regulators involved in TPB pathways have demonstrated a deep involvement of transcriptional regulation in TPB control during various processes of plant growth. The expression of key TPB genes is tightly coordinated with each other and with many PhANGs, with HY5 and GLKs possibly playing a central regulatory role. However, not all key TPB genes and coexpressed PhANGs are direct targets of HY5 and/or GLKs. Moreover, the classification of TPB genes into the four clusters based on light and circadian regulation is not strictly associated with the assignment of specific transcription factors to genes of each single cluster. The data suggest a complex regulation of these coexpressed genes by several transcription factors besides HY5 and GLKs under various conditions other than light and the circadian clock. Comprehensive promoter analysis of TPB genes and coexpressed PhANGs would provide further insight into the mechanism of coordinated regulation of these genes. Moreover, new molecular techniques have advanced our understanding of the involvement of chromatin remodeling and gene loci repositioning in the nucleus in transcriptional control. Thus, we anticipate an unraveling of the local regulation of TPB gene loci by specific transcription factors and also the global control of nuclear dynamics in response to endogenous and environmental stimuli. In addition, relationships between transcriptional regulation and other potent regulatory mechanisms such as post-translational control await elucidation.

As described here, most of the evidence for the TPB gene regulation in higher plants has come from *Arabidopsis* studies. However, besides common mechanisms conserved widely among plants, different types of plants could possess different regulatory systems specific to their life styles for regulating TPB genes. An increasing amount of large-scale transcriptome data for various types of plants will help reveal a picture of transcriptional regulation of TPB that is common and specific to plant species. Future molecular and biochemical studies in diverse plant species will also advance our understanding about the regulatory mechanisms of TPB genes in response to diverse developmental and environmental conditions.

## Author Contributions

KK conceived the review, collected data, and wrote the manuscript. TM helped to write the manuscript.

## Conflict of Interest Statement

The authors declare that the research was conducted in the absence of any commercial or financial relationships that could be construed as a potential conflict of interest.
